# A case series study on the effect of Ebola on facility-based deliveries in rural Liberia

**DOI:** 10.1186/s12884-015-0694-x

**Published:** 2015-10-12

**Authors:** Jody R. Lori, Sarah Danielson Rominski, Joseph E. Perosky, Michelle L. Munro, Garfee Williams, Sue Anne Bell, Aloysius B. Nyanplu, Patricia NM Amarah, Carol J. Boyd

**Affiliations:** Department of Health Behavior and Biological Sciences, University of Michigan, School of Nursing, 400 North Ingalls, Room 3352, Ann Arbor, MI 48109 USA; Global REACH, University of Michigan, Medical School, 234 Victor Vaughn Building, Ann Arbor, MI 48109 USA; Department of Orthopedic Surgery, University of Michigan, 109 Zina Pitcher Place, Ann Arbor, MI 48109 USA; Africare–Liberia, Congo Town, Opposite WAEC Office, 1000 Monrovia 10, Liberia

**Keywords:** Ebola, EVD, Facility delivery, Liberia, Maternal health

## Abstract

**Background:**

As communities’ fears of Ebola virus disease (EVD) in West Africa exacerbate and their trust in healthcare providers diminishes, EVD has the potential to reverse the recent progress made in promoting facility-based delivery. Using retrospective data from a study focused on maternal and newborn health, this analysis examined the influence of EVD on the use of facility-based maternity care in Bong Country, Liberia, which shares a boarder with Sierra Leone - near the epicenter of the outbreak.

**Methods:**

Using a case series design, retrospective data from logbooks were collected at 12 study sites in one county. These data were then analyzed to determine women’s use of facility-based maternity care between January 2012 and October 2014. The primary outcome was the number of facility-based deliveries over time. The first suspected case of EVD in Bong County was reported on June 30, 2014. Heat maps were generated and the number of deliveries was normalized to the average number of deliveries during the full 12 months before the EVD outbreak (March 2013 – February 2014).

**Results:**

Prior to the EVD outbreak, facility-based deliveries steadily increased in Bong County reaching an all-time high of over 500 per month at study sites in the first half of 2014 – indicating Liberia was making inroads in normalizing institutional maternal healthcare. However, as reports of EVD escalated, facility-based deliveries decreased to a low of 113 in August 2014.

**Conclusion:**

Ebola virus disease has negatively impacted the use of facility-based maternity services, placing childbearing women at increased risk for morbidity and death.

## Background

As we pass the one-year anniversary in early 2015 of the first reported death in Guinea from Ebola virus disease (EVD) the statistics are staggering. The World Health Organization reports the total number of cases attributed to EVD in West Africa has now surpassed 20,000 with the death toll nearing 8000 across the three most affected countries: Guinea, Sierra Leone and Liberia [1]. The most severe outbreak of EVD in history came to public awareness in March of 2014 [2] and quickly became a serious public health crisis.

In post-conflict countries such as Sierra Leone and Liberia, strained health care systems witnessed healthcare workers leaving their clinical posts – and patients shunning healthcare facilities [3]. In countries struggling with high rates of maternal and newborn mortality, the death of 366 health workers from EVD in Guinea, Sierra Leone, and Liberia [4] will have a significant impact on maternal and newborn outcomes [5]. Essential medical supplies and personnel have been diverted to Ebola Treatment Units, leaving facilities that provide delivery care without supplies or personnel [6]. Further, as the communities’ fear of EVD deepens and their trust in health providers weakens, women are choosing to deliver away from the health system’s reach, potentially reducing gains made in recent years to encourage facility delivery.

Prior to the EVD outbreak, facility-based deliveries with a skilled birth attendant were on the rise in rural Liberia [7]. Preliminary data from the 2013 Liberian Demographic Health Survey (LDHS) [8] show 96 % of women who gave birth in the five years preceding the survey received antenatal care from a skilled provider at least once during their last pregnancy, up from 79 % in 2007. Additionally, 56 % of births were reported as facility-based deliveries, a level much greater than the 37 % reported in the 2007 LDHS. The EVD crisis has the potential to reverse this progress.

This case series study explored how EVD has influenced utilization of facility-based delivery care in one rural county in Liberia. We used data collected between 2012 and 2014 from a large, USAID funded study designed to evaluate the effectiveness of maternity waiting homes (MWHs) in Bong County, Liberia, one of the areas most affected by the EVD. This USAID funded study allowed a longitudinal analysis of EVD’s effect on women’s use of facilities for childbirth in Bong County, Liberia. Indeed, the data provided an opportunity to examine the natural history of facility-based deliveries before and after the EVD and determine the effect of the EVD crisis on access to facility-based care during childbirth.

## Methods

This case series, examining the impact of EVD on rural facility-based delivery with a skilled birth attendant, was conducted through a retrospective record review of all deliveries reported at ten of 39 rural health care facilities and the two major referral hospitals in Bong County, Liberia. Institutional review board approval was obtained from the University of Michigan and the Liberia Ministry of Health and Social Welfare. Because this study used retrospective, de-identified data, informed consent was waived by the granting agencies.

### Setting

**Fig. 1:**
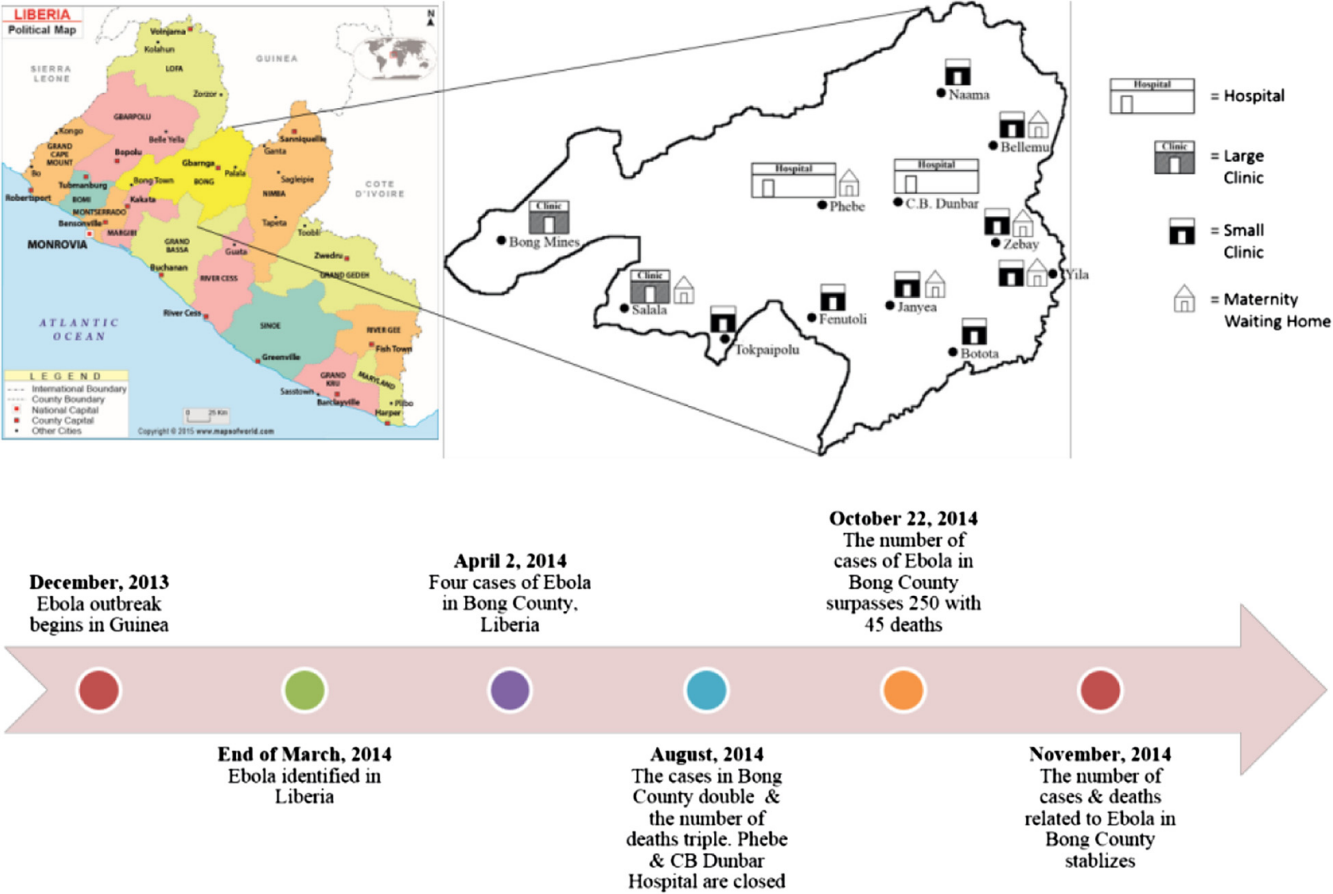
Timeline of Ebola outbreak in Bong County, Liberia. Cases refer to probable, suspected, or confirmed cases of Ebola. Deaths refer to probable, suspected, or confirmed deaths related to Ebola. Map used with permission from MapsofWorld.com

Bong County is one of 15 counties in Liberia with a population of approximately 330,000, making it one of the most populous counties in the country. It shares a common border with Guinea near the epicenter of EVD and where the same indigenous groups reside, moving freely across the two countries’ borders. The first suspected cases of EVD in Bong County were reported in June, 2014 [9] (see Fig. 1).

### Data collection and analysis

Between January 2012 and October 2014, data from the 12 study sites were collected from facility logbooks for the number of facility-based deliveries as well as from monthly reports generated by Liberian research assistants. Data collected on maternal and newborn indicators for the USAID funded study [7] allowed a retrospective determination of the number of women seeking facility-based delivery before and during the EVD crisis. Examples of the indicators collected for the USAID study include maternal sepsis, eclampsia, obstructed labor, post-partum hemorrhage, maternal deaths, neonatal sepsis, respiratory distress, apgars, stillbirth, and neonatal deaths.

Data on facility-based deliveries in Bong County were sorted and entered into Stata for analysis. Facility-based deliveries were sorted by hospital delivery, large clinics (catchment area over 20,000 population) and small clinics (catchment area under 20,000 population). All facilities are staffed by registered midwives. Both small and large clinics are equipped to provide basic emergency obstetric and newborn care. Hospitals are able to provide comprehensive obstetric emergency and newborn care.

To demonstrate the temporal change in the number of facility-based deliveries and MWH stays since the onset of the EVD outbreak, heat maps were generated for cumulative EVD cases, number of facility-based deliveries, and relative number of MWH stays in Bong County, Liberia. For purposes of constructing the maps, Bong County was divided by the estimated catchment area for each facility. The cumulative number of EVD cases in Bong County during each month since the onset of the outbreak was aggregated from Liberia Situation Reports generated by the Ministry of Health and Social Welfare. The number of facility-based deliveries and MWH stays were normalized to the average number of facility-based deliveries and MWH stays during the full 12 months before the EVD outbreak (March 2013 – February 2014) within the catchment area for each facility. A linear scale was used to generate all heat maps using a red-green-blue color scale.

## Results

Since 2012 and prior to the EVD outbreak, the use of facility-based deliveries steadily increased in Bong County, although seasonal fluctuations were apparent. In May 2014 the facility-based deliveries were at an all-time high at the study sites and appeared to be on an upward trajectory. In the first seven months of 2014 a combined total of 3436 facility-based deliveries were reported from study sites in Bong County, with average monthly facility-based deliveries between 400-500. However, as reports of EVD escalated, a nadir of facility-based deliveries occurred: in August there were only 113 facility-based deliveries and 160 in September. From July to August 2014, the number of EVD cases in Bong County tripled and the number of deaths began to rise (see Table 1). The two major referral hospitals were significantly scaled down when staff fled from a healthcare system ill equipped to handle the growing numbers of EVD patients. During this period, women’s utilization of facility-based deliveries was notably reduced.

**Table 1:** Facility-based deliveries, MWH stays, and Cumulative cases of EVD

2014	Jan	Feb	Mar	Apr	May	Jun	Jul	Aug	Sept	Oct
# of facility-based deliveries at study sites per month	443	404	524	548	587	520	410	113	160	306
# of MWH stays per month	55	38	75	84	84	68	59	27	30	32
Cumulative # of EVD cases in Liberia^a^	0	0	8	13	12^b^	51	329	1378	3458	6535
Cumulative # of EVD deaths in Liberia^a^	0	0	6	11	9^b^	34	156	694	1830	2413
Cumulative # of EVD cases in Bong County	0	0	0	0	0	1	39	128	273	440
Cumulative # of EVD deaths in Bong County	0	0	0	0	0	0	8	40	84	162

^a^retrieved from: http://www.cdc.gov/vhf/ebola/outbreaks/2014-west-africa/previous-case-counts.html

^b^the way in which Ebola cases and deaths were counted changed in May 2014

**Fig. 2:**
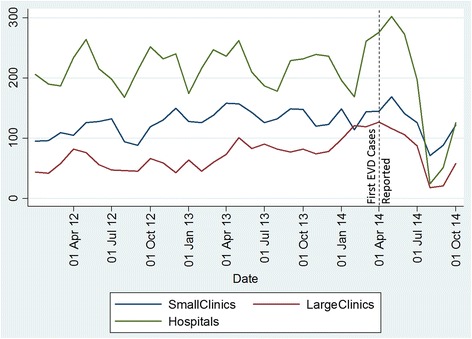
Trends in facility-based deliveries January 2012 to October 2014 by facility type. Figure 2 depicts trends before and after the Ebola outbreak in Bong County, Liberia at three types of facilities: small clinics (catchment area under 20,000 population); large clinics (catchment area over 20,000 population), and hospitals in the county

In October 2014, the two referral hospitals began to increase capacity and data indicate an upturn in facility-based delivery; unfortunately, despite an upturn, the numbers remain well below the pre-EVD increases in facility-based deliveries. Figure 2 depicts the steady rise in facility deliveries at small clinics, large clinics, and referral hospitals prior to the EVD outbreak and the rapid decline after EVD cases increased in Bong County, Liberia.

**Fig. 3:**
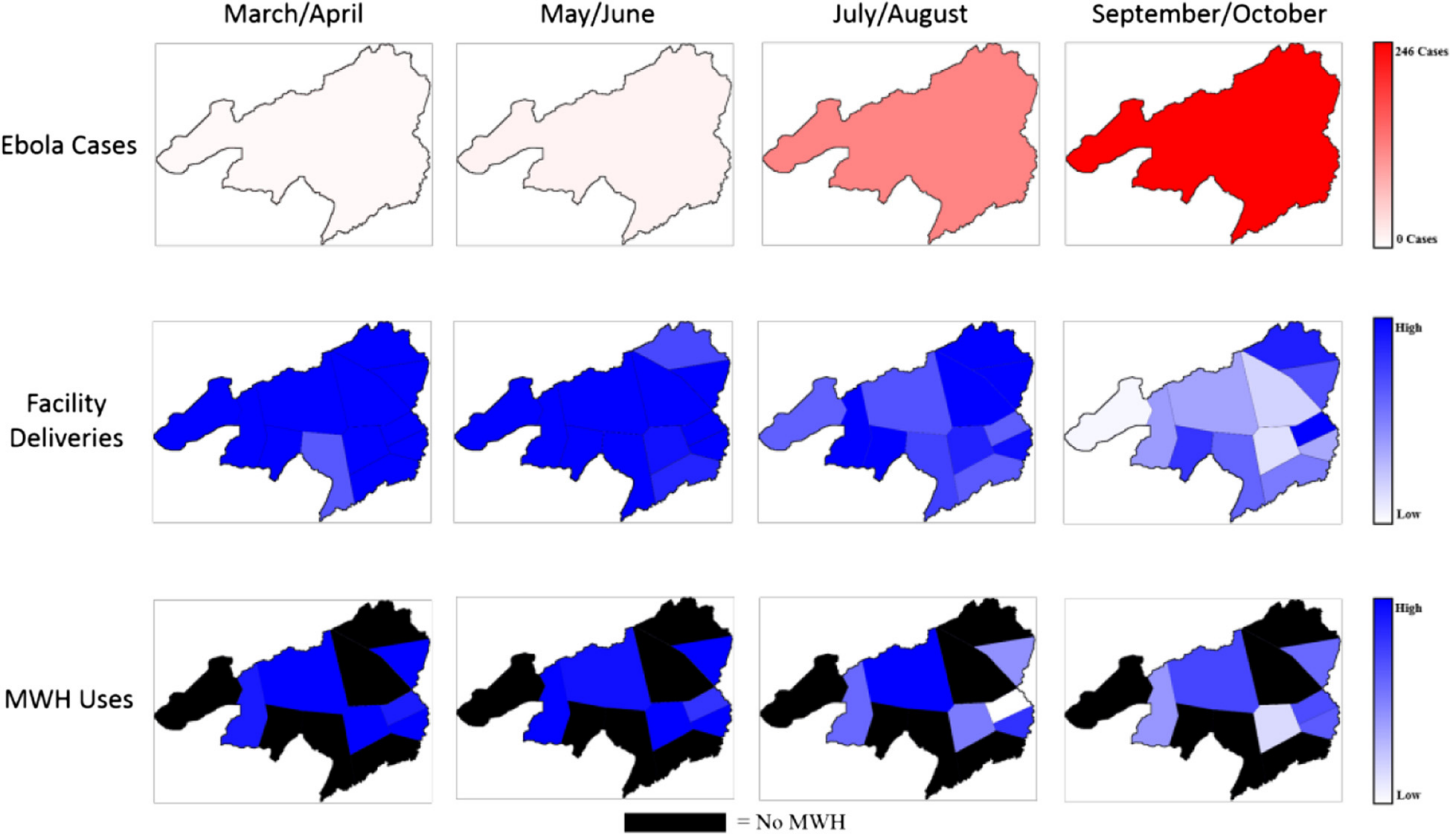
EVD cases, facility-based deliveries, and MWH stays in Bong County, Liberia (March 2014-October 2014) by catchment area for each clinic. Heat maps depict cumulative EVD cases, number of facility-based deliveries, and relative number of MWH stays in Bong County, Liberia

There were 440 EVD reported cases and 162 deaths in Bong County from January – October 2014. During this same time period, the number of facility-based deliveries per month in the study facilities decreased from a high of 587 in May to 113 in August. The number of MWH stays per month decreased from 84 to 27. During the first several months of the EVD outbreak in Liberia, Bong County was largely unaffected, reflected by the number of EVD cases and minimal decrease in facility-based deliveries and MWH use. However, as the disease spread the number of women delivering at a healthcare facility and those using MWHs decreased substantially (see Fig. 3).

## Discussion

Prior to the EVD outbreak Liberia had made significant progress in the number of women seeking facility-based care for childbirth. And while not enough time has elapsed to have a clear picture of how EVD will impact maternal and newborn health, preliminary evidence suggests the number of women seeking facility-based delivery dropped substantially during the height of the crisis. Multiple factors contributed to a decrease in facility-based deliveries during the EVD outbreak including: 1) a health system unprepared to care for large numbers of highly infectious patients, and 2) the major risk to staff for exposure to the Ebola virus during childbirth without the proper personal protective gear caused healthcare workers to remain at home rather than report to work and risk infection.

Fear among healthcare providers had a substantial impact on the accessibility and availability of facilities [6] as evidenced by significant reduction in services at the two major hospitals in Bong County during August and September in response to the EVD outbreak. By October 10, 2014 twenty-six cases of EVD and seven deaths were reported among healthcare workers in Bong County. While no hospitals or rural health care facilities completely closed in Bong County during the EVD outbreak, there was a substantial scale down in service provision at many facilities due to fear among staff of a disease that had already taken the lives of many healthcare providers. Although a general travel restriction was imposed in one section of the capital city, Monrovia, for a short period of time, healthcare providers were able to move from this area to work within healthcare facilities. No national or local policies mandated restrictions that impacted on health care workers or patient’s ability to reach healthcare facilities at any time during the EVD crisis.

The visible decline in the number of women seeking facility-based deliveries also highlights concerns about trust between patients and healthcare providers. Current media reports indicate a heightened distrust of health workers resulting in patients avoiding the health system [10–12]. At points throughout the EVD epidemic, the healthcare system was ill equipped to handle Ebola patients while also providing the “usual care” needed for maternity patients, malaria patients, and others [13]. Gaining trust is an essential component in the current response to Ebola [14]. Our past work in Liberia highlights the need for trust and teamwork among skilled providers, community health workers, and patients [7, 15, 16]. As we begin to examine the impact of EVD on maternal and newborn health it is imperative we consider how to rebuild trust in the healthcare system.

There are several limitations to this study including a short post EVD period and the use of retrospective pre-existing regional data. Additionally, the study design used is not optimal for making inferences about causality. Without data from deliveries that occurred outside facilities, this study was unable to correlate the decrease in the use of facility-based delivery services with poor maternal and newborn outcomes. Nonetheless, our data indicate what many have speculated – that EVD placed pregnant women and their unborn babies at increased risk for morbidity and death [17].

## Conclusion

This case series study was exploratory and designed to provide insights into the effect of the EVD outbreak on facility-based deliveries in Bong County, Liberia. Our findings highlight the collateral effect of EVD on maternal and newborn health and suggest that more sophisticated studies are needed to determine the many ways in which EVD is impacting maternal health.

## Abbreviations

EVD: Ebola virus disease
LDHS: Liberian Demographic Health Survey
MWH: Maternity waiting home

## References

[1] World Health Organization. Ebola response roadmap situation report. 2014. http://apps.who.int/iris/bitstream/10665/146763/1/roadmapsitrep_31Dec14_eng.pdf?ua=1&ua=1. Accessed 31 Dec 2014.

[2] Centers for Disease Control and Prevention. 2014 West Africa outbreak – initial announcement. 2014. http://www.cdc.gov/vhf/ebola/outbreaks/2014-west-africa/previous-updates.html. Accessed 4 Jan 2015.

[3] Dawson S. Exclusive: Liberia health system collapsing as Ebola spreads. Reuters. 2014. http://www.reuters.com/article/2014/08/07/us-health-ebola-liberia-idUSKBN0G72FC20140807. Accessed 17 Nov 2014.

[4] World Health Organization (2014). Ebola data and statistics.

[5] Hessou C. Liberia’s Ebola outbreak leaves pregnant women stranded*.* UNFPA. 2014. http://www.unfpa.org/public/home/news/pid/18139. Accessed 17 Nov 2014.

[6] Maron DF. Ebola strikes a blow against pregnant women and maternal care. Sci Am. 2014. http://www.scientificamerican.com/article/ebola-strikes-a-blow-against-pregnant-women-and-maternal-care/. Accessed 5 Jan 2015.

[7] Lori JR, Munro ML, Rominski S (2013). Maternity waiting homes and traditional midwives in rural Liberia. Int J Gynaecol Obstet.

[8] Liberia Institute of Statistics and Geo-Information Services (LISGIS). Liberia demographic and health survey 2013, preliminary report. 2013. https://dhsprogram.com/pubs/pdf/PR39/PR39.pdf. Accessed 4 Jan 2015.

[9] Ministry of Health and Social Welfare. National Ebola Response Update. 12 January 2015. http://www.mohsw.gov.lr/documents/Ebola%20Response%20Update_12%20January%202015.pdf. Accessed 11 June 2015.

[10] Biello D. Ebola exacerbates West Africa’s poverty crisis. Sci Am. 2014. http://www.scientificamerican.com/article/ebola-exacerbates-west-africa-s-poverty-crisis/. Accessed 5 Jan 2015.

[11] Boozary AS, Farmer PE, Jha AK (2014). The Ebola outbreak, fragile health systems, and quality as a cure. JAMA.

[12] Hayden E (2014). Ebola obstructs malaria control. Nature.

[13] Larsen SO. Other major diseases are going untreated in West Africa because of Ebola. neon tommy*,* Annenberg Digital News. 2014. http://www.neontommy.com/news/2014/10/other-major-diseases-are-now-going-untreated-west-africa-because-ebola. Accessed 16 Dec 2014.

[14] Preidt R. Trust is key to curbing West African Ebola outbreak, study finds. US News World Rep. 2014. http://health.usnews.com/health-news/articles/2014/11/17/trust-is-key-to-curbing-west-africa-ebola-outbreak-study-finds. Accessed 5 Jan 2015.

[15] Lori JR, Munro ML, Moore JE, Fladger J (2013). Lessons learned in Liberia: preliminary examination of the psychometric properties of trust and teamwork among maternal healthcare workers. BMC Health Serv Res.

[16] Lori JR, Wadsworth AC, Munro ML, Rominski S (2013). Promoting access: the use of maternity waiting homes to achieve safe motherhood. Midwifery.

[17] Hussain M. Ebola crisis putting pregnant women, infants lives at risk: UN. Reuters. 2014. http://www.reuters.com/article/2014/10/16/us-foundation-ebola-women-idUSKCN0I52FK20141016. Accessed 17 Nov 2014.

